# Investigating the impact of eating norms and collective autonomy support vs. collective control on unhealthy eating and its internalization

**DOI:** 10.1371/journal.pone.0276162

**Published:** 2022-10-19

**Authors:** Nada Kadhim, Catherine E. Amiot

**Affiliations:** Department of Psychology, Université du Québec à Montréal, Montréal, Québec, Canada; University of Macau, MACAO

## Abstract

Our eating behaviors are highly influenced by those of individuals surrounding us and the groups we belong to. The first goal of this experiment was to determine how social norms that encourage (pro-) vs. discourage (anti-) unhealthy eating influence people’s intentions and motivations to eat unhealthily. Since these norms can be conveyed by one’s group in a manner that either promotes group members’ autonomy (i.e., collective autonomy support), or pressures them into eating certain foods (i.e., collective control), the experiment also tests which of these types of messages promotes the highest conformity to group norms. Hence, the second goal of this experiment was to investigate this synergetic effect of pro- vs anti-unhealthy eating norms and of collective autonomy support vs. collective control on participants’ unhealthy eating intentions and their motivations for unhealthy eating. An experimental study (*N* = 341) using a 2 (eating norm: pro-unhealthy eating norm vs. anti-unhealthy eating norm) x 3 (type of group support: collective autonomy support vs. collective control vs. no support) design was conducted. Results showed that pro-unhealthy eating norms increased participants’ intentions to eat salty and fatty food, but also their amotivation (i.e., lack of motivation) for unhealthy eating relative to anti-unhealthy eating norms. In addition, when pro-unhealthy eating was encouraged in a controlling (vs. in an autonomy supportive) manner, participants reported higher intentions to eat tofu tacos. Finally, when pro-unhealthy eating was promoted by supporting group members’ autonomy, participants reported higher integrated regulation, i.e., a highly internalized motivation, for unhealthy eating. These results demonstrate that eating norms do not impact all types of unhealthy food consumption in the same manner, and that collective control may be motivating in uncertain contexts; furthermore, when individuals’ autonomy is supported and promoted by other group members, they are more susceptible to integrate unhealthy eating in their life.

## Introduction

Unhealthy eating and junk food consumption have adverse repercussions on health and well-being. Eating junk food–i.e., high energy density food that is rich in fat, sugar, and salt and which lacks nutritious elements [1, 2]–is not only associated with higher risks of developing physical dysfunctions such as obesity, diabetes, and heart diseases [2, 3], but also linked with increased psychological problems (e.g., depression [4, 5]; anxiety [6]).

In order to minimize these negative impacts on health, many social efforts and interventions have been designed to increase healthy eating and decrease unhealthy eating [7–9]. However, most of the time, eating takes place in the presence of others [10]. Thus, individuals are likely to turn to their eating partners and to important social groups in their lives (e.g., family, friends, colleagues) to obtain more proximal guidelines about what to eat. In fact, numerous studies have shown that social norms, which generally refer to the rules and principles that are shared within a social group [11], also influence eating behaviors [12, 13].

Importantly, the way these eating norms are encouraged and communicated within one’s social group should also matter. Eating partners and fellow group members can encourage other people within their group to eat certain foods by acknowledging and respecting their needs and desires; in other words, by being autonomy supportive [14]. However, these group members can also promote eating behaviors by pressuring others, i.e., by being controlling. Research is only beginning to investigate autonomy support vs. control in group contexts (c.f. [15]). As well, no research, to our knowledge, has investigated the impact of autonomy support and control in the context of unhealthy eating per se. Yet, doing so is important in order to capture how our proximal social groups can come to motivate us to eat unhealthily. To this aim, this research will investigate the impact of both pro- vs. anti- unhealthy eating norms, and test if the manner in which these norms are communicated by fellow ingroup members (either in an autonomy supportive vs. in a controlling manner) influences individual group members’ unhealthy eating intentions as well as their motivation for eating unhealthily (i.e., their degree of internalization of unhealthy eating; [16]).

### Social influences on eating and social norms

Various theoretical models have put forward different (and sometimes divergent) propositions to explain how the social context influences individuals’ eating behaviors. For example, while the impression management model [17] proposes that the presence of others *reduces* food intake, social facilitation theory [18] argues that individuals consume more food in the presence of others (also see modeling [19, 20], inhibitory model of social influence on eating [12]). Research on social norms allows to clarify these contradictory propositions by highlighting the importance of social groups and the fact that these groups may have their own specific eating guidelines and prescriptions. More specifically, studies have shown that descriptive norms, which refer to the actual behavior of a majority of individuals or group members in a specific context [21, 22], can either increase or decrease healthy and unhealthy eating behaviors, depending on what is specifically observed within the group (e.g. [23–26]). This impact of social norms on group members’ eating behaviors is coherent with the social identity approach (SIA; [27, 28]). Indeed, the SIA suggests that individuals are more likely to follow the norms of the social groups they belong to and value (also see [29]).

### Collective autonomy support vs. collective control

Much prior research has investigated the impact of social norms on individual group members’ eating behaviors [12, 13]. However, less research has focused on *how* these norms are communicated and conveyed to other ingroup members, and whether this influences the extent to which these norms will be followed. Self-determination theory (SDT; [30–33]) is employed here to specifically capture the manner in which normative information is communicated within a group. Based on SDT [30–33], when individuals’ need for autonomy is supported for a given behavior, they are then more likely to engage in this behavior frequently and voluntarily. A vast amount of SDT research has investigated the need for autonomy, along with how social contexts can promote this need [34]. Concretely, autonomy can be supported by being attentive to a person’s needs and beliefs, acknowledging their perspective, encouraging initiatives, and maximizing a sense of choice [14, 35]. In the presence of autonomy support, individuals feel the freedom to pursue their goals and endorse the values that are personally relevant to them. In contrast, a controlling context is characterized by a sense of pressure, the minimization of choice, and the use of a controlling language (e.g., “shoulds”, have to). As for norms, they can also be communicated either in a way to support the need for autonomy or thwart it.

Research conducted in the health context has shown that when people’s autonomy is supported (as opposed to thwarted), they are typically more likely to engage in healthy behaviors such as losing extra weight [36] and quitting smoking [37]. Yet, these beneficial consequences of autonomy support, as opposed to control, have mainly been investigated for positive and *health-promoting* behaviors. Research based on the SDT framework has only started to investigate the motivational processes that are involved when individuals engage in more harmful behaviors, that either cause harm to themselves or others (e.g., [34, 38]). Indeed, there is growing evidence showing that individuals can internalize harmful behaviors (e.g., consuming drugs, insulting someone) and engage in these behaviors for self-determined reasons [38–40]. Given the wide presence of unhealthy eating in our everyday lives and the importance that our eating partners have on this behavior [12, 13], it is highly relevant to apply SDT principles to understanding unhealthy eating as well.

#### Autonomy support vs. control at the group level

To specifically capture the role of groups in motivating people to eat unhealthily, and as another novel aspect, this research will investigate the impact of autonomy support vs. control when it originates from, and is communicated by, fellow ingroup members. Indeed, most research has investigated autonomy support vs. control at the interpersonal level of analysis, as opposed to the group level [35].

As notable exceptions, Dwyer and colleagues [41] investigated the role of group therapy in reducing anxiety and depression. In this study, individuals who felt that their autonomy was supported by other therapy group members experienced better therapy outcomes. Other studies, which experimentally manipulated autonomy support at the intergroup level, also showed that autonomy support from an outgroup can have important consequences such as improved intergroup relations [42; also see 15, 43]. Building on this prior work, in the present research, we specifically investigate the collective autonomy vs. collective control originating from an ingroup which is relevant to our participants in the context of unhealthy eating, namely, fellow university students [24, 44]. Doing so will allow to test if the manner in which a normative message is conveyed to group members with respect to their eating behaviors—either in an autonomy-promoting vs. controlling manner—will impact their behavioral intentions (i.e., to eat unhealthily and healthily) and their degree of internalization of unhealthy eating.

Given that collective autonomy support involves providing a rationale for a behavior and acknowledging the individual’s perspective, this type of support may further justify unhealthy eating, and thus amplify the impact of eating norms (see [39] for the facilitating role of legitimizing ideologies in the process of accepting and internalizing harmful group norms). In contrast, and given that collective control involves promoting a behavior by pressuring group members into adopting this behavior (i.e., without taking into consideration their needs, desires, and feelings) [14, 34], this type of support could weaken and reduce the influence of the eating norm. Indeed, previous research has shown that when group members are controlled and surveilled, this makes them less likely to personally and privately endorse the norms [45].

### Internalization of eating norms

Individuals’ personal reasons for following their groups’ norms can be diverse. The process through which individuals take social norms from external sources and make them their own is called internalization [16, 46]. When behaviors are internalized, they are followed autonomously and volitionally when given the choice, i.e., for self-determined reasons [47]. The internalization phenomenon has been extensively investigated from a SDT perspective [30–33]. According to SDT, motivations for one’s actions vary along a continuum, ranging from the more internalized and self-determined motives (i.e., intrinsic motivation), to the less self-determined ones (i.e., amotivation). Therefore, and in addition to measuring participants’ behavioral intentions to eat unhealthy and healthy foods, the current experiment will also assess their own motivations for eating unhealthily.

When applying the SDT continuum to the realm of unhealthy eating per se [48], the three self-determined motivations for eating unhealthy food include: intrinsic motivation (e.g., eating chocolate because it is inherently pleasurable to do so), integrated regulation (e.g., eating fast food because it reflects one’s lifestyle and values), and identified regulation (e.g., participating in pizza night with the family to reach an important goal, such as socially connecting with family members). The SDT continuum also consists of three non-self-determined motivations, which, when applied to unhealthy eating, include: introjected regulation (e.g., eating cake because of an internal sense of pressure and urge to do so), external regulation (e.g., eating junk food at a party to avoid negative comments such as being called “a dieter”), and amotivation (e.g., accepting the extra side of French fries offered by a restaurant for no particular reason). Because social norms have the power to normalize and legitimize even negative and harmful behaviors [11, 22, 39], social norms could hence promote the internalization of unhealthy eating, and increase individuals’ self-determined motivations for engaging in these behaviors. Socially, investigating these levels of internalization is important given that, when internalized, behaviors are then more likely to persist over the longer term [49]. Hence, understanding the social factors (i.e., eating norms and type of group support) that influence the internalization process could inform how we can prevent such behaviors from developing and from being internalized in the first place.

## The present experiment: Goals and hypotheses

The first goal of this experiment is to examine the impact of pro- vs. anti-unhealthy eating norms on individuals’ *unhealthy eating intentions* as well as on their degree of *internalization of unhealthy eating*. The experiment focused on university students, who are typically young adults and hence in a period where they are developing new eating habits [44]. As a result, they are establishing which points of reference will guide their eating behaviors. In addition, other fellow university students have been found to be a relevant and potent source of social influence on eating behaviors in this population [24, 50]. Also, it should be noted that university students, like most individuals, could be more likely to share meals with family and friends and as such, be influenced by their eating norms [12]. However, given that eating norms of these more proximal social groups are usually known, they are not easily amendable to experimental manipulation. Thus, the current research focused on the norms stemming specifically from fellow university students or, in other words, peers from the same university, as a shared ingroup. In line with the social norms literature, it is hypothesized that presenting participants with information showing that fellow university students endorse unhealthy eating (i.e., pro-unhealthy eating norm condition) will lead to higher intentions to eat unhealthily as well as to higher self-determined motivations for eating unhealthily (i.e., intrinsic motivation, integrated regulation, and identified regulation), as indicators of internalization, compared to being exposed to a norm that discourages unhealthy eating (i.e., anti-unhealthy eating norm condition).

As a second goal, the present experiment will test if this expected effect of social norms interacts with the manner in which these norms are communicated within the group (i.e., collective autonomy support vs. collective control) when predicting *unhealthy eating intentions* and the degree of *internalization of unhealthy eating*. Collective autonomy support and collective control will also originate from fellow university students as a relevant ingroup; these supports will be experimentally induced such that they will reinforce the manipulated student norm (either pro- or anti-unhealthy eating norm).

Based on the social norms and SDT literatures, it is expected that collective autonomy support will amplify the impact of pro-unhealthy eating norms. Concretely, in the *collective autonomy support condition*, participants in the pro-unhealthy eating norm condition should report higher unhealthy eating intentions, as well as higher self-determined motivations for eating unhealthily (i.e., intrinsic motivation, integrated regulation, and identified regulation), compared to those in the anti-unhealthy eating norm condition. In comparison, collective control is hypothesized to be demotivating and to attenuate the impact of pro-unhealthy eating norms; in the *collective control condition*, the expected difference in unhealthy eating intentions and self-determined motivations for unhealthy eating across the two eating norm conditions should hence be smaller.

To provide a baseline against which the collective autonomy support and the collective control condition can be compared, the current experiment also included a control condition, i.e., the *no support condition*, which provided no such group supports (i.e., and which tests the sole impact of norms). This condition will allow us to test the specific effects of pro- vs. anti-unhealthy eating norms on unhealthy eating intentions and their degree of internalization, unconfounded by the type of group support provided. In this no support condition, and in line with the social norms literature, a significant effect of social norms is expected. More specifically, unhealthy eating intentions and self-determined motivations for unhealthy eating will be higher in the pro-unhealthy eating norm condition compared to the anti-unhealthy eating norm condition. Yet, this effect of social norms should be smaller than in the autonomy support condition (i.e., which is expected to ‘amplify’ the impact of eating norms), and bigger than in the collective control condition (i.e., which is expected to ‘attenuate’ the impact of eating norms).

## Methods

### Participants and procedure

The sample size required for this experiment was determined using power calculations (G*Power 3.1.9.2, 2014; [51, 52]) based on a prior study by Wellen and colleagues [53] which used a similar experimental design and variables (i.e., independent variables: social identity, mood and norms; dependent variables: behaviors and behavioral intentions). At 80% power, α = .05, and *f* = 0.176, a sample size of 315 is required. In order to take into account potential outliers as well as other possible exclusions (e.g., participants who had guessed the goal of the experiment and those who did not pass the attention checks), 380 participants were recruited for this study. This experiment was approved by the *Institutional Ethics Committee for Research Involving Human Participants* (certificate #: 2863_e_2018), the ethics committee of the authors’ university. Data was collected between November 2018 and February 2019. Participants were told that their participation is voluntary. Written informed consent was obtained from all participants prior to completing the questionnaire. More precisely, undergraduate university students from the Business School of a French-speaking public university in Montreal were invited to complete the paper-and-pencil experiment in class. After receiving the study instructions, students who agreed to participate were randomly assigned to one of the experimental conditions. Random assignment was ensured by first using a random number generator, which generated multiple sets of numbers ranging from 1 to 6. Questionnaires were then placed using these numbers in piles prior to being distributed in class. Next, all participants who consented to participate completed the questionnaire during class under the supervision of the first author and at least one other trained experimenter. The questionnaire took between 20 and 30 minutes to complete. All statistical analyses were conducted using the IBM SPSS Statistics 22 software. It should also be noted that 2.51% of the data were missing from the entire dataset. These data were not missing completely at random (i.e., Little’s MCAR test: *χ*^*2*^(693) = 832.22, *p* < .001). Specifically, the majority of the missing values observable for variables assessed toward the end of the questionnaire. Since the percentage of missing data was below the 5% recommended threshold [54], no further action was taken to replace the missing data.

Some participants were excluded from the main analyses: four participants completed the questionnaire but failed to sign the consent form, one participant had participated in a similar study conducted by the same researchers, two participants had illnesses that could have affected their eating patterns (i.e., eating disorder, liver disease), 25 participants failed an attention check question. This multiple-choice format question tested whether participants had carefully read the information provided in the experimental manipulation (“Based on this survey, what is the proportion of students from [name of their university] who exceed the daily recommended intake of junk food? 1) 78% or 2) 22%”). In addition, three participants had univariate outlying scores (Z = ± 3.29) on at least two variables, two participants presented both univariate and multivariate outlier scores (Mahalanobis distance: *χ*^*2*^[27] = 55.48, *p*≤.001), and two other participants were solely multivariate outliers. The final sample consisted of 341 participants (female [F] = 63.3% women, male [M] = 36.7%). The mean age of participants was 23.71 (*SD* = 5.24, range: 18–47). Finally, their mean weight, height, and body mass index (BMI) were: 70.13 kg (*SD* = 17.41) [F = 63.62 kg (*SD* = 13.13), M = 81.31 kg (*SD* = 18.20)], 1.70 m (*SD* = 0.10) [F = 1.65 m (*SD* = 0.07), M = 1.79 m (*SD* = 0.06)] and 24.09 (*SD* = 5.14) [F = 23.37 (*SD* = 4.61), M = 25.33 (*SD* = 5.77)], respectively.

### Experimental design and protocol

Participants were randomly assigned to one of six experimental conditions following a 2 (eating norm: pro-unhealthy eating norm vs. anti-unhealthy eating norm) x 3 (type of group support: collective autonomy support vs. collective control vs. no support) design. The questionnaire was comprised of three sections. The first section consisted of sociodemographic questions and baseline variables (which will be detailed in the measures’ sections). The second section included the experimental manipulations. The first part of the experimental manipulations contained the *eating norm manipulation* and the second part, *the type of group support manipulation*, followed by questions about the manipulations (e.g., attention check, manipulation check). The last section of the questionnaire included the dependent variables. The questionnaire was presented to participants as being comprised of two distinct parts, one part which included the experimental manipulations and the other which included the dependent variables. This distinction was made in order to minimize demand characteristics and to conceal the actual goals of the study from the participants. The methodology file containing the experimental materials (i.e., consent form, questionnaires for each condition, debriefing form) as well as data and syntax files can be accessed via the following link: https://osf.io/kg5zd/?view_only=9290087f8d204e4b9f20399963359b59.

#### Eating norm manipulation

While both descriptive norms (i.e., the actual behavior of the majority of group members) and injunctive norms (i.e., behaviors that most group members value; [21, 22]) influence individuals’ eating behaviors, the impact of descriptive eating norms on eating behaviors has been found to be more consistent [55]. Thus, this experiment focused on manipulating descriptive norms (adapted from prior norm manipulations; [56, 57]) that either encourage vs. discourage unhealthy eating. In a broader perspective, while *pro-unhealthy eating norms* encourage “unhealthy” eating, *anti-unhealthy eating norms* should promote “healthy” eating. However, the use of the terms pro- vs. anti- terminology is employed here because the eating norm manipulation specifically focuses on unhealthy eating and junk food. This norm manipulation does not mention any type of “healthy food”.

The eating norm manipulation started with a definition of junk food. Specifically, and based on the World Health Organization [WHO; 2] guidelines, junk food was defined as food that is rich in salt, sugar or fat, or low in nutritional value [1]. Then, the daily maximum recommended intake of salt, fat and sugar by the WHO was provided. A few examples of junk food and the amount of junk food components they contain relative to the WHO recommendations were also provided to participants. For example, participants read that a standard chocolate tablet (e.g., a 4-finger milk chocolate KitKat [45 g]) includes almost all of the recommended amount of sugar and half of the amount of fat that should not be exceeded within one day. Participants were next presented with the results of a fictional survey containing specific manipulations of descriptive eating norms. They were told that students at their university (i.e., their ingroup; [24, 57]) were surveyed about the quantity of junk food they consume on a weekly basis. Graphs demonstrating the percentage of students who exceeded the daily recommendation of salt, sugar, and fat “most days of the week” vs. those who did not, were provided. Those in the *pro-unhealthy eating norm condition* were informed that 78% of their fellow students exceed these daily recommendations, while 22% do not exceed these daily recommendations. In the *anti-unhealthy norm condition*, these same percentages were used but were reversed.

#### Type of group support manipulation

The collective autonomy support and collective control manipulations were developed based on established conceptualizations and definitions of autonomy support and control, and adapted from prior studies [14, 58, 59]. After reading the eating norm manipulations, participants were presented with the type of support manipulation within the questionnaire. Specifically, they read that many students at their university who had completed the survey had also been ostensibly interviewed in the context of this prior survey. Then, participants in the collective autonomy support condition as well as those in the collective control condition were shown three written excerpts from these interviews.

The collective autonomy support and collective control conditions were always aligned with, and hence reinforced, the ingroup norm that had just been manipulated. Specifically, in the collective autonomy support condition, each excerpt represented one of the components of autonomy support [14, 58, 59], namely: 1) offering a rationale, 2) acknowledging participants’ perspective, and 3) fostering initiation and maximizing a sense of choice. In the collective control condition, the excerpts used to manipulate collective control maximized participants’ sense of pressure, minimized their choice, and used a controlling language [14, 58, 59]. It should be noted that participants in the no support condition only read the eating norm manipulation and were not presented with any texts or interview excerpts.

The original full texts used for the experimental manipulation can be found via the OSF link provided above (i.e., the study was conducted in French). For illustrative purposes, two segments are translated and presented here. The first segment was used in the collective autonomy support, pro-unhealthy eating norm condition. This segment focuses on fostering initiation and maximizing choice: “I eat junk food every now and then and like it a lot. I encourage other students to incorporate junk food into their diet and observe the positive consequences that doing so can have on our lives. However, we should not force ourselves to consume junk food or eat it impulsively or excessively, but do so when we feel the need to eat these types of food.” The second segment was used in the collective control, anti-unhealthy eating norm condition: “All kinds of junk food are bad. Believe me, I know what I’m talking about. So we should stop eating junk food and make this a habit. Personally, I have a very strict diet. I think we should all give up the habit of eating dessert every day, eat no more than two cubes of chocolate per week, and strictly avoid chips or fries at all costs.”

### Measures

#### Eating norm manipulation check

Participants responded to a question to ensure that they had read the eating norm manipulation carefully. The question was the following: “Based on this survey, to what extent do most of the students from [name of their university] exceed the daily recommendation of junk food?”. Participants provided their responses on a 7-point scale (1 = *not at all* to 7 = *absolutely*). It should be noted that a manipulation check for the type of group support manipulation was not included.

#### Baseline variables

A few baseline variables were measured prior to presenting the experimental manipulations to participants. First, three items measured the number of minutes of *light*, *moderate*, and *vigorous* physical activity engaged in during the previous week (adapted from the *International Physical Activity Questionnaire*; [60]). A few examples for each of these categories of exercise were given (e.g., light: walking to get to places like work, bus/subway or grocery store; moderate: swim gently; vigorous: cycling). Each of these three measures was used as a distinct variable. Second, one item measured participants’ usual *type of diet*. This measure focused on meat/animal product consumption, from least consumption to most (1 = vegan, 2 = vegetarian, 3 = pescatarian, 4 = flexitarian (consume meat occasionally), 5 = omnivore trying to reduce meat consumption, 6 = omnivore, 7 = other). Participants had to choose one of these options. In order to use responses to this question as a continuous variable (ranging from 1 = vegan to 6 = omnivore), six participants who chose “other” as their diet were excluded from the analysis focusing on this variable specifically. Third, three items were designed to measure the habitual frequency of consumption of each junk food component (i.e., foods that are rich in *sugar*, *fat* and *salt*). More specifically, participants were asked the following: “In general, how frequently do you consume food items that are rich in [sugar/fat/salt]?”. Responses were provided using the following Likert scale: 0 = “never”, 1 = “occasionally”, 2 = “once a week”, 3 = “2–3 times a week”, 4 = “4–6 times a week”, 5 = “once a day”, 6 = “twice a day”, 7 = “thrice a day”, 8 = “4 times in a day”, 9 = “5 or more times in a day”. Each of these three items was used as a separate variable.

#### Main dependent variables

As for the first block of dependent measures, which assessed unhealthy eating intentions, three categories of questions were developed for the purpose of the current experiment. Each of the individual unhealthy eating intention question presented below was used as a distinct variable in the analyses.

The first category measured *snack intentions*, which represents more habitual forms of eating [61]. Participants were asked to think about the next snack they were going to consume. Then, they were presented with four types of snacks: a cup of exotic fruits (e.g., mango, pineapple), chips, vegetables (e.g., carrots/celery/cucumber) and hummus, as well as chocolate. They were asked to indicate the likelihood of choosing each snack on a scale from 1 (*not likely at all*) to 7 (*very likely*). Then, participants were asked to choose only one snack (i.e., “If you had to choose only one of these snacks, which one would it be?”). A dichotomous variable representing relative unhealthy snack choice was created based on this question, where 0 represented the healthy options (i.e., vegetables and fruits) and 1 represented the unhealthy options (i.e., chips and chocolate).

The second category of unhealthy eating intentions measured participants’ *restaurant meal intentions;* i.e., their intentions to eat certain meals at a restaurant. Restaurant meals are more contextual and often represent punctual eating events for most individuals [62]. Specifically, participants were asked to imagine that they were invited to a restaurant for dinner that same night. Then, they were presented with four meal options. Each meal option was accompanied by the percentages of these meals’ junk food components in terms of the daily maximum recommendations by the WHO [2] (i.e., mac and cheese [typical two-cup serving = 90% of the daily recommended intake in salt, 2/3 of the daily recommended intake in fat], roasted salmon and white rice [1 serving = 14% of the daily recommended intake in salt, 13% of the daily recommended intake in fat], KFC® fried chicken breast [two chicken breasts = exceed the daily recommended intake in salt, 2/3 of the daily recommended intake in fat], and Cajun tofu tacos [1 serving = 34% of the daily recommended intake in salt, 23% of the daily recommended intake in fat]). Next, they were asked to indicate the likelihood of choosing each meal on a scale from 1 (*not likely at all*) to 7 (*very likely*). Similar to the snack intentions questions, participants were then asked to choose only one of the four meal options. Responses to this question were recoded on a 4-point scale, ranging from the healthiest meal options, to the unhealthiest options (i.e., salmon and rice = 1, tofu tacos = 2, mac and cheese = 3 and fried chicken = 4, respectively), to represent relative unhealthy restaurant choice. Note that this coding is directly based on the percentages of junk food components provided next to each restaurant option. This coding allows to objectively classify healthy vs. unhealthy restaurant options.

The third category of unhealthy eating intentions measures, i.e., *junk food components intentions*, assessed the three components (i.e., sugar, salt and fat) that are considered to constitute junk food [1, 2]. Specifically, and based on WHO guidelines, participants were presented with three questions, which specifically asked whether they intended to exceed the daily recommended intake of *sugar*, *salt* and *fat* within the next week, respectively (i.e., 1) “How many days in the next week do you intend to exceed the daily recommended intake of *sugar*?”, 2) “How many days in the next week do you intend to exceed the daily recommended intake of *salt*?, and 3) “How many days in the next week do you intend to exceed the daily recommended intake of *fat*?”). After each of these questions, participants were provided with examples of the maximum amount of the daily recommendation of each junk food component: sugar (2/3 of a can of coke OR 2 scoops of ice cream), salt (Hamburger trio at McDonald’s® restaurant [i.e., BLT burger sandwich, Parmesan garlic fries, coke] + small Caesar salad OR about half of a bag of Doritos® nacho family size chips), and fat (1 KFC® fried chicken breast OR 1 McDonald’s® bacon and egg McMuffin®). Responses on these three questions were recorded on a scale from 0 (*no day*) to 7 (*everyday*).

*Motivations for unhealthy eating intentions*, representing the second block of dependent measures, were assessed using six items [48]. These measures have been adapted from different established motivations scales [32, 63] to the specific context of unhealthy eating. More specifically, participants were asked to think about their intentions to eat foods rich in salt, sugar, and fat and the reasons why they will consume these foods in the upcoming week. Then, they were presented with the following six motivation items: intrinsic motivation (i.e., “because I derive satisfaction from consuming these foods”), integrated regulation (i.e., “because consuming these foods is part of my lifestyle”), identified regulation (i.e., “because I think it is important to consume such foods”), introjected regulation (i.e., “because I would feel bad if I don’t consume these foods”), external regulation (i.e., “because other people close to me insist that I consume these foods”), and amotivation (i.e., “I do not know; I really do not have any good reasons for consuming such foods”). Responses on these items were recorded on a scale from 1 (*not at all*) to 7 (*extremely*). To fully capture SDT’s motivation continuum, each of the six motivation items was used as a distinct dependent variable.

#### Exploratory dependent variable: Perceptions of norm conflict

Given the existence of broader societal recommendations that promote pro-healthy eating (e.g. [2, 7, 8]), the manipulated pro-unhealthy eating norm, in particular, could be seen to conflict with these recommendations. Thus, an exploratory question measured participants’ perceptions of norm conflict. More specifically, this question assessed perceptions of conflict between the eating behaviors of university students and the broader norms concerning eating (i.e., “I feel that the survey results diverge from the general dietary recommendations”). Responses were rated on a 7-point scale (1 = *not at all* to 7 = *absolutely*).

It should be noted that participants also completed three additional items on perceptions of norm conflict. However, since the Cronbach alpha was weak for the overall 4-item scale (α = .21), only the most relevant item was used for the analyses. Measures of participants’ psychological well-being and their perceptions of the normality of unhealthy eating were also included at the end of the questionnaire as exploratory variables; however, the majority of the effects observed on these measures were not significant and did not contribute to the main research questions. The description of these additional measures and the results observed are presented in the S1 File.

## Results

### Preliminary analyses

#### Differences on baseline variables

Given that our hypotheses were theoretically grounded and planned a priori, an alpha level of .05 was used in our analyses [64, 65]. First, a series of 2 (eating norm: pro-unhealthy eating norm vs. anti-unhealthy eating norm) X 3 (type of group support: collective autonomy support vs. collective control vs. no support) ANOVAs were conducted on the BMI, amount of physical activity (i.e., light, moderate and vigorous physical activity), type of diet and habitual unhealthy eating intentions measures (i.e., sugar, fat and salt intentions). These analyses allowed to ensure that participants in the different experimental conditions did not differ on any of these baseline measures, taken prior to the manipulations. Indeed, participants who lead a more “healthy lifestyle” are also more likely to eat healthily and engage in physical activity, as the two main components of a healthy lifestyle [66, 67].

Neither the main effects of eating norms and type of group support nor their interactions were significant (i.e., eating norm main effect: *F*s ranged from 0.005 to 3.80, *p*s ranged from .052 to .942, *η*_*p*_^2^s ranged from .000 to .011; type of group support main effect: *F*s ranged from 0.023 to 3.02, *p*s ranged from .050 to .977, *η*_*p*_^2^s ranged from .000 to .018; Eating Norm X Type of Group Support interaction: *F*s ranged from 0.01 to 1.714, *p*s ranged from .182 to .993, *η*_*p*_^2^s ranged from .000 to .010). These results confirm that there were no pre-existing differences in BMI, amount of physical activity, type of diet and habitual unhealthy food consumption across the different experimental conditions.

#### Eating norm manipulation check

A 2 (eating norm: pro-unhealthy eating norm vs. anti-unhealthy eating norm) X 3 (type of group support: collective autonomy support vs. collective control vs. no support) ANOVA was conducted on the eating norm manipulation check question. A significant main effect of the eating norm, *F*(1, 318) = 814.61, *p* < .001, η_p_^2^ = .719, revealed that participants in the pro-unhealthy eating norm condition perceived that other students at their university ate more unhealthy food (*M* = 6.12, *SD* = 0.79) than those in the anti-unhealthy eating norm condition (*M* = 2.82, *SD* = 1.25). The main effect for type of group support on perceptions of unhealthy food consumption was not significant, *F*(2, 318) = 0.57, *p* = .569, η_p_^2^ = .004, nor was the interaction between eating norm and type of group support, *F*(2, 318) = 1.44, *p* = .238, η_p_^2^ = .009. These results indicate that as expected, perceptions of unhealthy food consumption were higher among participants in the pro-unhealthy eating norm condition than those in the anti-unhealthy eating norm condition. Thus, the eating norm manipulation elicited the expected perceptions of unhealthy food consumption among our participants.

### Main analyses

#### Future unhealthy eating intentions

A series of 2 (eating norm: pro-unhealthy eating norm vs. anti-unhealthy eating norm) X 3 (type of group support: collective autonomy support vs. collective control vs. no support) ANOVAs were conducted to assess the impact of eating norms and type of group support on participants’ intentions to eat snacks, restaurant meals, and junk food components.

The ANOVAs conducted on the *snack intentions measures* (i.e., fruits, chips, vegetables, chocolate and the relative unhealthy snack choice) showed that, contrary to hypotheses, neither the main effects of eating norms and type of group support nor the interactions between eating norms and type of group support were significant (see Table 1 for results).

**Table 1:** Comparisons of unhealthy eating intentions across conditions.

Variable	Condition						F_norm_ (η_p_^2^)	F_group support_ (η_p_^2^)	F_int_ (η_p_^2^)
	Pro-unhealthy eating norm			Anti-unhealthy eating norm					
	Collective autonomy support *M*(*SD*)	Collective control *M*(*SD*)	No support *M*(*SD*)	Collective autonomy support *M*(*SD*)	Collective control *M*(*SD*)	No support *M*(*SD*)			
**Snack intentions**									
**Fruits**	4.97 (1.98)	5.14 (1.81)	4.77 (1.91)	5.09 (2.03)	5.21 (1.71)	5.43 (1.65)	1.98 (.006)	0.17 (.001)	0.86 (.005)
**Chips**	2.74 (1.92)	2.64 (1.73)	2.75 (1.48)	2.35 (1.60)	2.65 (1.77)	2.64 (1.94)	0.75 (.002)	0.23 (.001)	0.38 (.002)
**Vegetables**	4.34 (1.85)	4.64 (2.04)	4.53 (2.01)	4.57 (2.03)	4.52 (1.85)	4.22 (2.06)	0.09 (.000)	0.31 (.002)	0.54 (.003)
**Chocolate**	3.07 (1.92)	3.79 (1.97)	2.86 (1.81)	2.84 (1.94)	3.29 (1.95)	3.38 (1.80)	0.11 (.000)	2.80 (.017)	2.16 (.013)
**Relative unhealthy snack choice**	0.15 (0.36)	0.21 (0.41)	0.19 (0.40)	0.12 (0.33)	0.17 (0.38)	0.17 (0.38)	0.49 (.001)	0.61 (.004)	0.01 (.000)
**Restaurant meal intentions**									
**Mac and cheese**	3.28 (2.17)	3.21 (1.99)	3.25 (1.88)	3.29 (1.84)	3.83 (2.12)	3.30 (2.15)	1.07 (.003)	0.52 (.003)	0.78 (.005)
**Salmon and rice**	4.66 (2.05)	4.69 (2.30)	4.77 (2.02)	4.63 (2.09)	4.40 (2.30)	5.16 (1.89)	0.01 (.000)	1.21 (.007)	0.73 (.004)
**KFC® fried chicken**	2.22 (1.77)	2.66 (1.97)	2.79 (1.82)	2.25 (1.73)	2.44 (1.96)	2.40 (1.76)	0.93 (.003)	1.32 (.008)	0.37 (.002)
**Tofu tacos**	4.28 (2.30)	3.74 (2.27)	4.19 (2.21)	3.81 (2.24)	4.77 (2.12)	4.32 (2.04)	0.90 (.003)	0.36 (.002)	3.22* (.019)
**Relative unhealthy restaurant choice**	1.82 (0.97)	1.89 (1.13)	1.89 (1.03)	1.81 (1.01)	1.85 (0.82)	1.70 (0.87)	0.57 (.002)	0.19 (.001)	0.29 (.002)
**Junk food components intentions**									
**Sugar**	2.40 (1.52)	2.34 (2.08)	2.40 (1.62)	2.32 (1.73)	2.29 (1.70)	2.24 (1.57)	0.26 (.001)	0.02 (.000)	0.03 (.000)
**Salt**	2.44 (1.61)	1.97 (1.97)	2.37 (1.68)	1.64 (1.47)	1.90 (1.34)	1.79 (1.61)	7.26** (.021)	0.25 (.001)	1.42 (.009)
**Fat**	2.07 (1.57)	2.36 (2.02)	2.44 (1.84)	1.75 (1.42)	1.88 (1.42)	1.67 (1.62)	8.17** (.024)	0.45 (.003)	0.53 (.003)

*p < .05

**p < .01.

The ANOVAs conducted on participants’ *restaurant meal intentions* for the next meal (i.e., mac and cheese, salmon and rice, KFC® fried chicken, tofu tacos, relative unhealthy restaurant choice) revealed that, contrary to our predictions, none of the main effects of the eating norms or type of group support were significant (see Table 1 for results). A significant interaction was however observed between eating norms and type of group support on tofu tacos intentions, a healthy restaurant meal option. Interpretation of this interaction revealed that in the collective control condition, participants presented with the pro-unhealthy eating norm had lower intentions to eat tofu tacos (*M* = 3.74, *SD* = 2.27) than those presented with the anti-unhealthy eating norm (*M* = 4.77, *SD* = 2.12), *F*(1, 329) = 5.76, *p* = .017, η_p_^2^ = .017. However, in the collective autonomy support condition, there was no difference in intentions to eat tofu tacos between participants presented with the pro-unhealthy eating norm compared to those presented with the anti-unhealthy eating norm, *F*(1, 329) = 1.31, *p* = .254, η_p_^2^ = .004. Similarly, in the no support condition, participants presented with the pro-unhealthy eating norm did not differ in their intentions to eat tofu tacos relative to those presented with the anti-unhealthy eating norm, *F*(1, 329) = 0.09, *p* = .766, η_p_^2^ = .000. These results indicate that contrary to expectations, the effect of the pro-unhealthy eating norm manipulation was amplified in the collective control condition, rather than in the collective autonomy support condition. Other interactions on restaurant meal intentions were not significant.

The ANOVAs conducted on the *junk food components intentions* (i.e., sugar, salt and fat) revealed a main effect of eating norms on salt and on fat. As expected, participants in the pro-unhealthy eating norm condition reported higher intentions to eat food rich in salt in the next week (*M* = 2.26, *SD* = 1.76) compared to those in the anti-unhealthy eating norm condition (*M* = 1.77, *SD* = 1.48; see Table 1). Similarly, participants in the pro-unhealthy eating norm condition had higher intentions to eat food rich in fat (*M* = 2.29, *SD* = 1.82) compared to those in the anti-unhealthy eating norm condition (*M* = 1.76, *SD* = 1.49). However, contrary expectations, the main effect of eating norms on sugar was not significant. Also, the main effects of type of group support were not significant. In addition, none of the interactions between eating norms and type of group support on food that is rich is salt, fat and sugar were significant (see Table 1 for results). Thus, eating norms increased conformity specifically for intentions to eat food that is rich in salt as well as fat.

#### Motivations for unhealthy eating

A series of 2 (eating norm: pro-unhealthy eating norm vs. anti-unhealthy eating norm) X 3 (type of group support: collective autonomy support vs. collective control vs. no support) ANOVAs were conducted to assess the impact of eating norms and type of group support on the six types of *motivations for unhealthy eating* (see Table 2 for results). A significant main effect of eating norms on amotivation was revealed. More specifically, participants in the pro-unhealthy eating norm condition reported higher levels of amotivation for unhealthy eating (*M* = 4.18, *SD* = 2.23) than those in the anti-unhealthy eating norm condition (*M* = 3.52, *SD* = 2.20). None of the other main effects of eating norms nor of type of group support were significant (Table 2).

**Table 2:** Comparisons of motivations to eat unhealthy food across conditions.

Variable	Condition						F_norm_ (η_p_^2^)	F_group support_ (η_p_^2^)	F_int_ (η_p_^2^)
	Pro-unhealthy eating norm			Anti-unhealthy eating norm					
	Collective autonomy support *M*(*SD*)	Collective control *M*(*SD*)	No support *M*(*SD*)	Collective autonomy support *M*(*SD*)	Collective control *M*(*SD*)	No support *M*(*SD*)			
**Intrinsic motivation**	4.97 (1.93)	4.49 (1.84)	4.89 (1.64)	4.67 (1.70)	4.85 (1.88)	4.86 (1.93)	0.00 (.000)	0.36 (.002)	0.88 (.005)
**Integrated regulation**	3.45 (1.74)	2.87 (1.77)	2.72 (1.56)	2.70 (1.48)	3.21 (1.91)	2.93 (1.62)	0.13 (.000)	0.73 (.004)	3.51* (.021)
**Identified regulation**	2.00 (1.45)	1.65 (1.16)	1.65 (1.16)	1.80 (1.35)	1.52 (0.90)	1.88 (1.37)	0.06 (.000)	1.67 (.010)	0.97 (.006)
**Introjected regulation**	1.50 (1.03)	1.40 (0.89)	1.54 (0.87)	1.65 (1.16)	1.56 (1.18)	1.81 (1.47)	2.49 (.008)	0.83 (.005)	0.09 (.001)
**External regulation**	1.91 (1.43)	1.53 (1.17)	1.60 (1.00)	1.62 (1.18)	1.89 (1.32)	1.45 (0.86)	0.04 (.000)	1.35 (.008)	2.29 (.014)
**Amotivation**	4.15 (2.29)	4.61 (2.25)	3.77 (2.10)	3.71 (2.32)	3.34 (2.25)	3.49 (2.05)	7.38** (.022)	0.80 (.005)	1.52 (.009)

*p < .05

**p < .01.

A significant Eating Norm X Type of Group Support interaction on the integrated regulation also emerged. Interpretation of this interaction showed that, as expected, in the collective autonomy support condition, participants presented with the pro-unhealthy eating norm had higher levels of integrated regulation for unhealthy eating (*M* = 3.45, *SD* = 1.74) compared to those presented with the anti-unhealthy eating norm (*M* = 2.70, *SD* = 1.48), *F*(1, 325) = 5.72, *p* = .017, η_p_^2^ = .017. However, in the collective control condition, there was no difference in levels of integrated regulation between participants presented with the pro-unhealthy eating norm and those presented with the anti-unhealthy eating norm, *F*(1, 325) = 1.03, *p* = .311, η_p_^2^ = .003. Similarly, in the no support condition, participants presented with the pro-unhealthy eating norm did not differ in their levels of integrated motivation for unhealthy eating compared to those presented with the anti-unhealthy eating norm, *F*(1, 325) = 0.46, *p* = .499, η_p_^2^ = .001. Contrary to expectations, the interactions between eating norms and type of support on the two other self-determined motivations (i.e., intrinsic motivation and identified regulation) were not significant. Also, the other interactions on non-self-determined motivations were not significant.

### Exploratory analysis

#### Perceptions of norm conflict

A 2 (eating norm: pro-unhealthy eating norm vs. anti-unhealthy eating norm) X 3 (type of group support: collective autonomy support vs. collective control vs. no support) ANOVA was conducted on participants’ perceptions of norm conflict to explore the impact of eating norms and type of group support on this measure. A significant main effect of norm was found on perceptions of norm conflict, *F*(1, 319) = 56.41, *p* < .001, η_p_^2^ = .150. Participants in the pro-unhealthy eating norm condition perceived higher levels of norm conflict (*M* = 4.54, *SD* = 2.24) than those in the anti-unhealthy eating norm condition (*M* = 2.92, *SD* = 1.65). While the main effect of type of group support on perceptions of norm conflict was not significant, *F*(2, 319) = 2.45, *p* = .088, η_p_^2^ = .015, the Eating Norm X Type of Group interaction on perceptions of norm conflict was significant, *F*(2, 319) = 3.86, *p* = .022, η_p_^2^ = .024. Interpretation of this interaction revealed that in the collective autonomy support condition, participants presented with the pro-unhealthy eating norm perceived higher levels of norm conflict (*M* = 5.16, *SD* = 1.91) than those presented with the anti-unhealthy eating norm (*M* = 2.92, *SD* = 1.63), *F*(1, 319) = 36.28, *p* < .001, η_p_^2^ = .102. The same difference was observed in the collective control condition (i.e., in which participants presented with the pro-unhealthy eating norm also perceived higher levels of conflict [*M* = 4.54, *SD* = 2.33] than those presented with the anti-unhealthy eating norm [*M* = 2.71, *SD* = 1.61], *F*[1, 319] = 22.37, *p* < .001, η_p_^2^ = .066) and in the no support condition (i.e., such that participants presented with the pro-unhealthy eating norm also perceived higher levels of conflict [*M* = 3.89, *SD* = 2.32] than those presented with the anti-unhealthy eating norm [*M* = 3.07, *SD* = 1.72], *F*[1, 319] = 4.97, *p* = .026, η_p_^2^ = .015). However, the difference in means appears particularly pronounced in the collective autonomy support as well as in the collective control conditions, compared to the no support condition; hence explaining the significant interaction effect observed.

## Discussion

The first objective of this research was to examine how social norms pertaining to unhealthy eating impact individuals’ intentions to eating unhealthily and the degree to which they personally accept and internalize unhealthy eating. As a second objective, and in line with emerging research that integrates group processes and SDT’s postulates (e.g. [15, 42]), the current study combined these approaches by investigating the role of autonomy support and control stemming specifically from an ingroup (i.e., collective autonomy support vs. collective control) as factors that can amplify vs. hinder, respectively, the impact of eating norms on group members’ intentions to eat unhealthily and their motivation for doing so.

Two main blocks of dependent variables were assessed in the current experiment, namely participants’: 1) unhealthy eating intentions and 2) their motivations for (i.e., degree of internalization of) unhealthy eating. Whereas the first part of the discussion section focuses on the results pertaining to the impact of unhealthy eating norms and of collective autonomy support vs. collective control on *unhealthy eating intentions*, the second part focuses on their impact on the degree of *internalization of unhealthy eating*.

### The impact of eating norms on unhealthy eating intentions

Pro-unhealthy eating norms were expected to increase unhealthy eating intentions compared to anti-unhealthy eating norms. This expected effect of norms was only found on two measures of eating intentions, namely on participants’ intentions to consume foods that are rich in salt as well as fat. These results confirm that when a relevant social group holds pro-unhealthy eating norms, its group members are more likely to increase their intentions to eat unhealthy types of foods such as those rich in salt and in fat.

However, this main effect of pro vs. anti-unhealthy eating norms was not observed on participants’ intentions to eat food that is rich in sugar. This finding could be explained by the fact that societal norms and guidelines regarding the unhealthy nature of sugar and its adverse consequences (e.g., weight gain, diabetes) are often communicated to the public by the media and are quite clear [68]. Hence, it might have been difficult to go against this consensus with a punctual, situationally-salient experimental manipulation that promotes eating sugary food. Instead, these chronically salient societal recommendations may have incited participants to report low intentions to eat food rich in sugar, even when they were experimentally exposed to a pro-unhealthy eating norm. In contrast, for salty and high-fat food, on which the norms manipulation did have an effect, it is possible that a gray area exists. For example, the highly popular ketogenic diet encourages the consumption of foods that are low in carbohydrates and high in fat [69, 70]. Some low-fat products are also criticized and considered to be less healthy or equally as unhealthy as their full-fat counterparts [71]. There are also varying beliefs on the potential benefits of different types of salt (e.g., Himalayan vs. table salt; [72]). These diverse and changing information individuals are exposed to might make fatty and salty food more compelling than sugary food. Thus, in this context, the experimentally-induced pro-unhealthy eating norms might have been easier to endorse specifically for foods that are rich in salt and fat (compared to food rich in sugar).

Also contrary to our expectations, eating norms did not impact any of the snack eating intentions measures. This pattern of findings could be explained by the fact that snacking can be seen as a habitual behavior [61]. Thus, snack consumption intentions–given they are more habitual and possibly more engrained in people’s lifestyles–might also have been less influenced by the situational experimental manipulation employed in the current study. Furthermore, eating norms did not result in significant main effects on the restaurant meal intentions; as it will be detailed in the section just below, it is only when eating norms and group support were simultaneously presented that one significant effect was observed on the restaurant intentions measures. Research in the eating behaviors domain also shows that individuals consume more food when more food choices are available [73–75]. These prior findings could help to explain why more effects for norms were observed on the three measures referring to junk food components intentions. Indeed, these three measures were general and referred to each broad category of junk food (i.e., these measures did not refer to a particular type of food). For these measures, participants had the freedom to imagine which specific kinds of foods they could refer to. Hence, they might have perceived more food options when reporting their junk food component intentions. In comparison, the restaurant meal and snack intentions measures, although they were diverse, referred to very specific types of food. In this sense, participants might have perceived them as more restrictive than junk food components intentions and felt more constrained/perceived less choice. As a result, and in line with the food availability literature [73–75], participants might have endorsed the snack and restaurant meals to a lesser extent than junk food components given the reduced choices available.

### The synergetic effect of eating norms and type of group support on unhealthy eating intentions

In terms of the expected interactions between pro-unhealthy eating norms and collective autonomy support, one significant finding was observed on intentions to eat tofu tacos. However, the specific shape and interpretation of this interaction did not support our hypothesis. Precisely, we found that in the collective control condition, pro-unhealthy eating norms decreased participants’ intentions to eat tofu tacos, as a healthy option, relative to the anti-unhealthy eating norm. This unexpected facilitating role of collective control in increasing unhealthy eating on this measure find echo in the results of some prior studies that had revealed a more nuanced role for autonomy and control, and which contrasts with the SDT view. These prior studies had shown that autonomy support is not always motivating for everyone, and that control does not consistently impede motivation among all individuals. For example, and in line with the person-environment fit theory [76–78], research has shown that employees who have a non-self-determined orientation, as opposed to a self-determined orientation, experience lower stress in organizational environments that offers them less autonomy and freedom in their work [79].

In the context of the current experiment, and in line with these prior findings, we might have specifically created a situation in which collective control, as opposed to collective autonomy support, actually increased the impact of the pro-unhealthy eating norm on people’s desire to eat the more unhealthy restaurant choices. Indeed, collective control might have accentuated the effect of the pro-unhealthy eating norm condition because norm conflict was possibly induced when the pro-unhealthy eating norm was manipulated. More specifically, mentioning WHO’s daily maximum recommendations for consuming each of the junk food components (i.e., salt, sugar and fat) in the experimental instructions possibly activated a healthy norm that opposes the pro-unhealthy eating student norm. Indeed, this intuition about the presence of higher norm conflict in the pro-unhealthy eating norm condition was confirmed with the exploratory analysis on the perceptions of norm conflict reported above.

As a result, the presence of these competing norms possibly made the message promoted by the ingroup, which encouraged unhealthy eating, particularly ambiguous; individuals in the pro-unhealthy eating norm condition may also have felt particularly confused and uncertain as to which eating norm to follow. In this ambiguous context created in the pro-unhealthy eating norm condition, it is possible that pro-unhealthy eating norms that were communicated in a controlling manner were also perceived by participants as being clearer, authoritative, and capable of reducing their uncertainty. This could explain why it was actually the collective control condition, as opposed to the collective autonomy support condition, that accentuated the impact of pro-unhealthy norms on participants’ intentions to eat unhealthy restaurant meals. Indeed, social psychological research has shown that uncertainty motivates individuals to seek guidance from social groups [80–82]. The motivating role of collective control in an uncertain situation also aligns with uncertainty reduction theory [81], which proposes that when individuals experience uncertainty, they become more likely to identify with a social group that has clear, unambiguous, and well-defined guidelines/norms, and to conform to these norms. This process in turn reduces the initial unpleasant feeling of ambiguity and allows individuals to gain control and clarity [81, 82].

### The impact of eating norms on self-determined and non-self-determined motivations

As for the second block of dependent variables, i.e., motivations for unhealthy eating, the only significant effect of the eating norms was observed on amotivation for unhealthy eating. However, the specific direction observed for this effect was also not expected. Precisely, participants who were exposed to a norm encouraging unhealthy eating were more uncertain about their reasons for unhealthy eating (i.e., higher levels of amotivation) compared to those who were exposed to a norm discouraging unhealthy eating. It is possible that the norm conflict present in the pro-unhealthy eating condition and the uncertainly it appears to have created among our participants, may also have made individuals doubt the very reasons why they engage in unhealthy eating. Hesitation with regards to one’s behavior is indeed a defining component of amotivation.

The expected main effects of eating norms on the three self-determined motivations (i.e., intrinsic motivation, integrated regulation, identified motivation for unhealthy eating) were also not confirmed. In terms of intrinsic motivation, it is possible that in the context of unhealthy eating, this type of motivation is especially relevant and applicable, given the pleasurable taste of junk food and the positive experience that individuals generally have while consuming it. Hence, intrinsic motivation for eating unhealthy food might remain consistently high and possibly be less amenable to change following a short-term experimental induction. Indeed, the means of intrinsic motivation for unhealthy eating were particularly high in all conditions (overall *M* = 4.79 on a 7-point Likert scale, *SD* = 1.82). A supplementary repeated-measures ANOVA (with motivation type as the within-participant factor) also revealed that intrinsic motivation was significantly and consistently higher than the five other motivation types. The high level of intrinsic motivation is coherent with the findings of another study also examining various types of motivations for unhealthy eating. Specifically, this prior study had also found that intrinsic motivation is more endorsed than all other motivation types [48].

As for identified regulation, this type of self-determined motivation appears to be generally less directly applicable in the context of unhealthy eating compared to the two other forms of self-determined motivations. Indeed, the overall mean observed for identified regulation was particularly low (*M* [*SD*] = 1.76 [1.25], range *M* [*SD*] = 1.52 [0.90] to 2.00 [1.45]) across conditions. Given the strong presence of pro-healthy eating messages in our societies, it is possible that although individuals eat unhealthy food, they do not consider this as being a behavior that they find “important”. This lower applicability of the identified regulation to unhealthy eating, combined with the strong presence of pro-health messages in our societies, may explain why this type of regulation was also less susceptible to change following a short-term, punctual experimental manipulation that promotes unhealthy eating per se. As for integrated regulation, it is only when pro-unhealthy eating norms were conjointly presented with collective autonomy support that an increase in this type of regulation was observed. This interaction will be further explained in the next section.

### The synergetic effect of eating norms and type of group support on the self-determined motivations

The expected interaction effect between eating norms and type of group support on the self-determined motivations emerged specifically on integrated regulation for unhealthy eating, a self-determined form of motivation that represents a quite high level of internalization [31, 33]. Precisely, and as expected, when collective autonomy support was present, participants presented with the pro-unhealthy eating norm had higher levels of integrated regulation for unhealthy eating compared to those presented with the anti-unhealthy eating norms. High levels of integrated regulation indicate that a behavior (i.e., unhealthy eating) is part of one’s values, habits, and lifestyle. This result confirms that a pro-unhealthy eating norm conveyed in a manner that supported group members’ autonomy was successful in promoting this quite engrained type of eating regulation among our participants. However, the interaction effect of eating norms and type of group support was not observed on intrinsic motivation and identified regulation. Again, these results could be explained by the fact that intrinsic motivation may be quite entrenched with respect to unhealthy eating and less likely to be modified by punctual experimental manipulations, and by the lower general relevance and applicability of the identified regulation in the context of unhealthy eating.

### Theoretical and practical implications

#### Theoretical implications

As mentioned previously, research stemming from SDT [30–33] mainly focuses on the internalization process and the consequences of autonomy support vs. control in the context of positive and healthy behaviors (e.g., healthy eating, engaging in physical exercise). In fact, according to classic SDT principles [30–33], when basic psychological needs, i.e., the needs for autonomy, competence and relatedness, are satisfied, individuals are more likely to internalize and engage in positive behaviors that allow for personal growth. However, according to a classic SDT perspective, more negative behaviors that could cause harm to the self or others are more likely to be engaged in when basic psychological needs are thwarted. Thus, such behaviors should not be internalized nor stem from self-determination. In contrast, the social norms perspective based on the SIA [27, 28, 83] suggests that when individuals belong to a group and identify with this group, they are more likely to internalize and follow its norm, regardless of the valence of the behavior encouraged and promoted.

Some of the current results could be interpreted in light of these two competing perspectives. Contrary to expectations, collective autonomy support did not amplify nor facilitate the impact of eating norms on any of the unhealthy eating intentions variables. Thus, in line with classic SDT principles [30–33], it is possible that collective autonomy support was less strongly activated given the focus, in the current study, on unhealthy eating (as opposed to healthy eating), a behavior that could actually harm the individual and impede their personal growth. In addition, the collective control manipulation per se strengthened the impact of norms and increased intentions to eat tofu tacos. This result might also indicate that the pressure and control individuals experienced in this experimental condition impeded the satisfaction of their need for autonomy and further encouraged the selection of an unhealthy eating option, as a more ‘negative’ or detrimental food choice. As for the results regarding the synergetic effect of collective autonomy support and eating norms on degree of internalization, similar to unhealthy eating intentions, the absence of significant effects on the more internalized motivations (i.e., intrinsic motivation and identified regulation) supports classic SDT principles. Specifically, collective autonomy support for a potentially detrimental behavior, and for a norm which is incoherent with the general (pro-healthy) societal norm, might have impeded the process of internalization, which should be more likely present for healthy eating. However, the increase in integrated regulation for unhealthy eating is in line with the social norms and the SIA approach. Specifically, collective autonomy support for the ingroup norm, even a potentially harmful one, encouraged and promoted integrated regulation, a highly internalized motivation. In sum, both theoretical perspectives with regards to internalization and the pursuit of unhealthy eating seem useful to account for the present results. Future research should investigate the specific instances and mechanisms in which internalization of negative and harmful behaviors are possible. In fact, recent theoretical advances and empirical studies have highlighted the simultaneous contradictory yet complementary nature of the SDT as well as social norms and SIA perspectives in understanding harmful behaviors (see [39]).

#### Practical implications

The present findings also inform us on possible health-promoting interventions. First, and as seen above, only a few significant effects of eating norms were observed. These results suggest that the punctual and manipulated pro-unhealthy eating norm may not have been potent enough to go against the general societal norm encouraging healthy eating, and which is promoted in the interventions put forward by governments and other institutions. On the basis of the current findings, it is possible that the presence of such pro-healthy eating messages could serve to counteract the pro-unhealthy eating norms that are promoted by other social groups. Second, as reported above and in line with the SDT perspective, the current findings regarding eating intentions suggest that collective autonomy support may be particularly difficult to activate for unhealthy eating per se, a behavior that could impede personal growth and health. Thus, health-promoting interventions should continue to focus on encouraging more healthy eating along with autonomy support.

Finally, the findings regarding the increase observed in integrated regulation in the presence of both collective autonomy support and pro-unhealthy eating norms can also inform the design of health-supporting interventions. Specifically, and in line the social norms/SIA perspective, these findings show the danger of supporting autonomy for a potentially negative behavior when it is coherent with individuals’ lifestyles and system of values. Hence, interventions should adopt a preventive focus (and occur as early as possible in a person’s life) in order to change problematic eating before other potentially harmful habits such as physical inactivity are also adopted. In this vein, interventions encouraging healthy eating or a more generally healthy lifestyle, should be multifaceted and target multiple aspects related to health (e.g., more healthy eating, more physical activity, healthy sleep habits), to ensure congruence across all life domains, which is also a defining element of integrated regulation.

### Limitations and future directions

Integrating the social norms and self-determination theory literatures, the experimental nature of this research allowed us to investigate the respective and combined impacts of eating norms and type of group support on participants’ intentions to eat unhealthily and their degree of internalization of these behaviors. Even though the present study employed an experimental design, its cross-sectional nature could present some limitations. For example, the cross-sectional design used does not allow us to examine the long-term effects of the manipulations. In addition, the hypothetical nature of the measures employed and the fact that they focused on eating intentions per se did not allow us to measure actual nor observed dietary intake. Although behavioral intentions are significantly linked to actual behavior [84], future research could precisely measure eating behaviors in the laboratory. Previous research has shown that eating norms predict both eating intentions and eating behaviors [25, 26, 85]. However, measuring actual dietary intake in the presence of both eating norms and collective autonomy support vs. collective control would shed light on whether the internalization process of unhealthy eating leads to actual changes in behavior in this theoretically and practically important life context. Also, in terms of eating intentions and motivations for unhealthy eating, the original plan was to conduct fewer analyses, on five global variables (i.e., global unhealthy snack intentions; global unhealthy restaurant intentions; global junk food intentions; self-determined motivation for unhealthy eating; non-self-determined motivation for unhealthy eating). However, given the unreliable alphas observed for most of these global variables, analyses were conducted on the individual items, and more analyses than planned were hence conducted. The current findings should be replicated with more reliable measures.

Additionally, based on the WHO recommendations for consuming salt, sugar and fat, we provided participants with a valid cut-off for determining what is considered healthy vs. unhealthy eating. However, since providing this information may have activated a norm conflict between these WHO recommendations and the ingroup norm manipulation that just followed, future studies should systematically compare a condition where these general pro-health societal recommendations are presented, to a condition where they are not mentioned. Yet, it is possible that the broad salience and importance of pro-healthy eating norms in society generally make the experimental manipulation of pro-unhealthy eating norms difficult and not completely plausible, regardless of the ‘pureness’ of the eating norm manipulation employed herein. This investigation highlights the importance of taking these broader societal health-promoting norms directly into account when investigating unhealthy norms and behaviors. More generally, this chronic salience of pro-healthy eating norms may explain why relatively few significant effects involving the unhealthy eating norm manipulation were observed in the current study. In itself, and from a public health perspective, this general observation can be seen as encouraging.

Moreover, some of the questions measuring eating intentions might have biased participants’ responses. More specifically, providing examples from fast food chains such as KFC® and McDonald’s® had the advantage of specifying the concrete amounts of junk food elements from restaurants most individuals are familiar with. However, and given the possible negative connotation associated with fast food chains, individuals might have been primed to think about these types of food when considering their intentions to eat junk food. Thus, individuals who had intentions to eat foods with similar amounts of junk food components as those of the fast-food restaurants might have been less likely to report high intentions to consume these foods. Thus, future research could refrain from referring to specific fast-food chains when measuring unhealthy eating. Also, levels of social identification with the group that promotes eating norms as well as the degree to which this group was valued by participants were not directly measured in the present research. It is possible that participants who identified less strongly with the ingroup targeted in the current research (i.e., fellow university students), and those who valued this ingroup to a lesser extent, were also less impacted by the eating norm manipulation. Future research could directly test whether individuals who identify more strongly with their ingroup will be more likely to follow the pro vs. anti-unhealthy eating, in line with SIA [27, 28, 83].

## Conclusion

In sum, the current findings demonstrated that pro-unhealthy eating group norms were successful in increasing participants’ intentions to consume some unhealthy foods (i.e., foods rich in salt and fat). Also, participants who were exposed to a pro-unhealthy eating norm and for whom this norm was communicated in a controlling manner by a relevant ingroup had higher intentions to eat tofu tacos. This motivating role of collective control in promoting unhealthy eating was possibly due to the presence of an induced conflict between the manipulated pro-unhealthy eating norm and the broader pro-health societal guidelines and norms surrounding eating. More precisely, collective control, as opposed to collective autonomy support, by being clear, directive, and authoritative, may have been able to reduce the state of uncertainty created by the normative conflict particularly present in the pro-unhealthy eating norm condition. Furthermore, this normative conflict and the uncertainty it possibly created among our participants, may have increased their doubts about unhealthy eating and led them to question this behavior (i.e., higher amotivation). Finally, collective autonomy support, in conjunction with the pro-unhealthy eating, increased integrated regulation for unhealthy eating per se, thus demonstrating the amplifying impact of autonomy support for this highly internalized motivation. Clearly, various theoretically and socially important questions remain to be answered to expand our understanding of the role of norms that promote vs. discourage unhealthy eating, as well as the manner in which these norms are communicated to group members.

## Supporting information

S1 File (DOCX)
