# Supplementary material for: Investigating the impact of eating norms and collective autonomy support vs. collective control on unhealthy eating and its internalization
Source: PLoS One. 2022 Oct 19;17(10):e0276162. doi: 10.1371/journal.pone.0276162 (PMC9581406; doi:10.1371/journal.pone.0276162)
Supplement: S1 File — (DOCX) [file pone.0276162.s001.docx]

**Supplementary analyses**

**Exploratory measures and analyses: Comparisons of supplementary measures across conditions**

**Psychological well-being**

Some additional and exploratory psychological well-being variables were also included at the end of the questionnaire. Specifically, participants’ situational well-being was assessed with the following measures: the International Positive and Negative Affect Scale ([1b]; Positive Affect α=.73; Negative Affect α=.69), the Subjective Vitality Scale ([2b]; α=.88), the State Anxiety Scale ([3b-4b]; α=.72), the Single-Item Self-Esteem Scale [5b], and a five-item depression scale created by the authors based on the emotions typically associated with depressive states (Diagnostic and Statistical Manual of Mental Disorders [DSM-5; 6b]); given the weak reliability of this measure [α=.28], analyses were performed on each item individually.

A series of 2 (eating norm: pro-unhealthy eating norm vs. anti-unhealthy eating norm) X 3 (type of group support: collective autonomy support vs. collective control vs. no support) ANOVAs were conducted on these well-being variables (see S1 Table for results).

Only one statistically significant result was found. More specifically, a significant Eating Norm X Type of Group Support effect was found on negative affect. Interpretation of this interaction revealed that in the collective autonomy support condition, participants presented with the pro-unhealthy eating norm experienced higher levels of negative affect (*M*=2.16, *SD*=1.07) compared to those presented with the anti-unhealthy eating norm (*M*=1.61, *SD*=0.75), *F*(1, 324)= 9.15, *p*=.003, η_p_^2^=.027. However, in the collective control condition, participants presented with the pro-unhealthy eating norm did not differ in their levels of negative affect compared to those presented with the anti-unhealthy eating norm, *F*(1, 324)= 0.22, *p*=.643, η_p_^2^=.001. Similarly, in the no support condition, there was no difference in negative affect among participants presented with the pro-unhealthy eating norm relative to those presented with the anti-unhealthy eating norm, *F*(1, 324)= 0.00, *p*=.931, η_p_^2^=.000. All other effects were non significant (please see S1 Table below).

**Normality of eating junk food**

Another exploratory measure included at the end of our questionnaire assessed participants’ perceptions of the extent to which it can sometimes be normal or appropriate to eat junk food. This scale consisted of four items developed by the authors (i.e., “I find it appropriate to eat junk food on certain occasions (e.g., Christmas, birthdays, holidays)”, “When I am with certain groups of people (e.g., my family, my friends), I allow myself to eat a certain amount of junk food”, “I think it's okay to eat junk food in some settings (e.g., on weekends, at restaurants)”, “I think it's okay to eat junk food sometimes, especially if you don't have time to cook.”; α=.76). This measure allowed to explore whether eating norms and type of group support affected participants’ perceptions that it is normal to eat junk food consumption in certain contexts. A 2 (eating norm: pro-unhealthy eating norm vs. anti-unhealthy eating norm) X 3 (type of group support: collective autonomy support vs. collective control vs. no support) ANOVA revealed that neither the main effects of eating norm, the type of group support, nor their interaction influenced the extent to which eating junk food is perceived as normal within certain contexts (see S1 Table for results).

**S1 Table. Comparisons of supplementary measures across conditions.**

| **Variable** | **Condition** | | | | | | **F_norm_ (η_p_^2^)** | **F_group support_ (η_p_^2^)** | **F_int_ (η_p_^2^)** |
| --- | --- | --- | --- | --- | --- | --- | --- | --- | --- |
|  | **Pro-unhealthy eating norm** | | | **Anti-unhealthy eating norm** | | |  |  |  |
|  | **Collective autonomy support**  ***M*(*SD*)** | **Collective control**  ***M*(*SD*)** | **No support**  ***M*(*SD*)** | **Collective autonomy support**  ***M*(*SD*)** | **Collective control**  ***M*(*SD*)** | **No support**  ***M*(*SD*)** |  |  |  |
| Negative affect | 2.16 (1.07) | 1.76 (0.91) | 1.94 (0.89) | 1.61 (0.75) | 1.85 (1.13) | 1.92 (1.01) | 2.23 (.007) | 0.46 (.003) | 3.46* (.021) |
| Positive affect | 3.79 (1.40) | 3.54 (1.45) | 3.96 (1.24) | 3.78 (1.26) | 3.70 (1.40) | 4.13 (1.18) | 0.53 (.002) | 2.93 (.018) | 0.17 (.001) |
| Subjective vitality | 3.92 (1.30) | 4.30 (1.36) | 4.18 (1.22) | 4.15 (1.41) | 4.15 (1.35) | 4.40 (1.21) | 0.49 (.001) | 1.18 (.007) | 0.72 (.004) |
| State anxiety | 2.87 (1.08) | 2.85 (1.17) | 2.79 (1.16) | 2.62 (0.95) | 2.68 (1.10) | 2.94 (1.05) | 0.53 (.002) | 0.39 (.002) | 1.07 (.007) |
| Confident^R^ | 3.91 (2.00) | 3.53 (2.00) | 3.11 (1.52) | 3.48 (1.90) | 3.32 (1.92) | 3.42 (1.65) | 0.28 (.001) | 1.60 (.010) | 1.22 (.007) |
| Self-critical | 4.04 (2.13) | 3.84 (2.14) | 4.02 (2.11) | 3.71 (2.10) | 3.73 (2.17) | 4.14 (2.09) | 0.19 (.001) | 0.56 (.003) | 0.31 (.002) |
| Concentrated^R^ | 3.63 (1.72) | 3.66 (1.67) | 3.58 (1.44) | 3.59 (1.78) | 3.77 (1.86) | 3.30 (1.45) | 0.15 (.000) | 0.78 (.005) | 0.39 (.002) |
| Tired | 4.53 (2.05) | 3.96 (2.16) | 4.79 (1.69) | 4.28 (1.90) | 4.35 (2.00) | 4.30 (2.05) | 0.28 (.001) | 1.04 (.006) | 1.42 (.009) |
| Sad | 2.12 (1.65) | 1.79 (1.36) | 2.00 (1.48) | 1.75 (1.31) | 1.58 (0.90) | 2.23 (1.59) | 0.57 (.002) | 2.51 (.015) | 1.37 (.008) |
| Self-esteem | 4.60 (1.72) | 4.63 (1.74) | 5.04 (1.22) | 4.54 (1.62) | 4.74 (1.45) | 4.33 (1.81) | 1.49 (.005) | 0.19 (.001) | 1.97 (.012) |
| Normality of eating junk food | 5.06 (1.26) | 4.73 (1.63) | 4.76 (1.32) | 4.59 (1.17) | 5.07 (1.05) | 4.59 (1.32) | 0.49 (.002) | 0.86 (.005) | 2.66 (.016) |

**p* < .05

^R^These items were reverse-coded so that higher scores on these items indicate higher levels of depressive symptoms.

**Supplementary analyses: The role of gender and BMI**

Given the fact that gender [7b-8b] and BMI [9b] can play a role on eating habits, some exploratory additional exploratory analyses were conducted to examine the role of gender and BMI on the main variables. First, ANOVAs with gender as the independent variable and unhealthy eating intentions (Table S2) as well as motivations to eat unhealthy food (Table S3) as the dependent variables were conducted. As shown in Table S2, women reported higher intentions to consume fruits as well as vegetables, while men reported higher intentions to eat food rich in sugar, salt, and fat. This pattern of results aligns with prior research showing that compared to men, women tend to consume more fruit and vegetables [7b] and less junk food (e.g., [8b]). In addition, and as shown in Table S3, men reported higher levels of integrated and identified regulation for unhealthy eating. Specifically, men show a higher tendency to find that consumption of junk food is part of their lifestyle or that it is important to them; men hence reported higher levels of internalization of unhealthy eating. Combined with the observation that in this sample, men also reported higher intentions to consume the junk food components, our findings more broadly align with the SDT literature, which showed that higher levels of internalization are associated with higher levels of behavioral frequency [49].

Next, ANCOVAs, with gender as the covariate, eating norms and type of support as independent variables and unhealthy eating intentions (Table S4) as well as motivations to eat unhealthy food (Table S5) as the dependent variables were conducted. The pattern of results observed in these additional analyses was the same as the pattern observed in the analyses presented in the manuscript per se. In sum, although some gender differences in terms of intentions to eat some types of food and degree of internalization of unhealthy eating were observed in the current sample, these differences do not alter the general pattern of the results; furthermore, these additional findings align with prior literature.

Finally, ANCOVAs, with BMI as a covariate, eating norms and type of support as the independent variables, and unhealthy eating intentions (Table S6) as well as motivations to eat unhealthy food (Table S7) as the dependent variables. As can be seen in Tables S6 and S7, most of the results reported in the manuscript were replicated in these additional analyses. A few differences can be noted. Specifically, a main effect for type of support on intentions to eat chocolate was observed. However, and given that collective autonomy support and collective control are specifically provided for the given eating norm, the main effect of type of support (without taking into account the type of norm) is not theoretically interpretable. In addition, in these supplementary ANCOVAs, the interaction effects between eating norms and type of support on intentions to eat tofu tacos and on integrated regulation for unhealthy eating became non-significant.

**S2 Table. Comparisons of unhealthy eating intentions as a function of gender.**

| **Variable** | **Gender** | | **F_gender_ (η_p_^2^)** |
| --- | --- | --- | --- |
|  | **Male**  ***M*(*SD*)** | **Female**  ***M*(*SD*)** |  |
| **Snack intentions** | | | |
| **Fruits** | 4.68 (1.95) | 5.33 (1.76) | 9.97** (.029) |
| **Chips** | 2.79 (1.76) | 2.53 (1.73) | 1.69 (.005) |
| **Vegetables** | 4.17 (1.94) | 4.64 (1.97) | 4.58* (.013) |
| **Chocolate** | 3.18 (1.80) | 3.22 (1.98) | 0.04 (.000) |
| **Relative unhealthy snack choice** | 0.21 (0.41) | 0.15 (0.35) | 2.24 (.007) |
| **Restaurant meal intentions** | | | |
| **Mac and cheese** | 3.31 (1.89) | 3.36 (2.09) | 0.04 (.000) |
| **Salmon and rice** | 4.64 (2.08) | 4.78 (2.12) | 0.33 (.001) |
| **KFC® fried chicken** | 2.70 (1.86) | 2.32 (1.80) | 3.38 (.010) |
| **Tofu tacos** | 4.10 (2.16) | 4.21 (2.24) | 0.19 (.001) |
| **Relative unhealthy restaurant choice** | 1.86 (1.05) | 1.81 (0.93) | 0.26 (.001) |
| **Junk food components intentions** | | | |
| **Sugar** | 2.62 (1.84) | 2.17 (1.60) | 5.63* (.016) |
| **Salt** | 2.48 (1.77) | 1.76 (1.51) | 15.95** (.045) |
| **Fat** | 2.40 (1.83) | 1.82 (1.56) | 9.32** (.027) |

**p* < .05

***p* < .01

Please note that the Levene’s tests for homogeneity of variances were significant on the following variables: relative unhealthy snack choice, mac and cheese, intentions to eat food rich in sugar, intentions to eat food rich in salt and intentions to eat food rich in fat, suggesting that the variances across experimental conditions are heterogenous for these variables. However, the uncorrected statistics are reported in the present table for all of the variables, given that the statistics that correct for heterogeneity of variances do not yield the η_p_^2^s, and that these corrected statistics revealed the same pattern of findings for these variables (in terms of the *p*s and *F*s observed).

**S3 Table.** **Comparisons of motivations to eat unhealthy food as a function of gender.**

| **Variable** | **Gender** | | **F_gender_ (η_p_^2^)** |
| --- | --- | --- | --- |
|  | **Male**  ***M*(*SD*)** | **Female**  ***M*(*SD*)** |  |
| **Intrinsic motivation** | 4.72 (1.79) | 4.83 (1.83) | 0.32 (.001) |
| **Integrated regulation** | 3.33 (1.80) | 2.78 (1.59) | 8.24** (.024) |
| **Identified regulation** | 2.06 (1.50) | 1.59 (1.06) | 11.20** (.033) |
| **Introjected regulation** | 1.71 (1.16) | 1.51 (1.09) | 2.49 (.008) |
| **External regulation** | 1.69 (1.16) | 1.64 (1.18) | 0.10 (.000) |
| **Amotivation** | 3.75 (2.31) | 3.93 (2.19) | 0.51 (.002) |

**p* < .05

***p* < .01

Please note that the Levene’s tests for homogeneity of variances were significant on identified regulation for unhealthy eating, suggesting that the variances across experimental conditions are heterogenous for this variable. However, the uncorrected statistics are reported in the present table for all of the variables, given that the statistics that corrected for heterogeneity of variance do not yield the η_p_^2^s, and that these corrected statistics revealed the same pattern of findings for these variables (in terms of the *p*s and *F*s observed).

**S4 Table. Comparisons of unhealthy eating intentions across conditions while controlling for gender.**

| **Variable** | **Condition** | | | | | | **F_gender_ (η_p_^2^)** | **F_norm_ (η_p_^2^)** | **F_group support_ (η_p_^2^)** | **F_int_ (η_p_^2^)** |
| --- | --- | --- | --- | --- | --- | --- | --- | --- | --- | --- |
|  | **Pro-unhealthy eating norm** | | | **Anti-unhealthy eating norm** | | |  |  |  |  |
|  | **Collective autonomy support**  ***M*(*SE*)** | **Collective control**  ***M*(*SE*)** | **No support**  ***M*(*SE*)** | **Collective autonomy support**  ***M*(*SE*)** | **Collective control**  ***M*(*SE*)** | **No support**  ***M*(*SE*)** |  |  |  |  |
| **Snack intentions** | | | | | | | | | | |
| **Fruits** | 4.96 (0.24) | 5.17 (0.24) | 4.78 (0.24) | 5.11 (0.25) | 5.23 (0.27) | 5.38 (0.24) | 9.47** (.028) | 1.81 (.005) | 0.24 (.001) | 0.70 (.004) |
| **Chips** | 2.74 (0.22) | 2.63 (0.23) | 2.75 (0.23) | 2.34 (0.23) | 2.64 (0.25) | 2.66 (0.23) | 1.82 (.005) | 0.71 (.002) | 0.26 (.002) | 0.43 (.003) |
| **Vegetables** | 4.33 (0.25) | 4.67 (0.26) | 4.53 (0.26) | 4.59 (0.26) | 4.54 (0.28) | 4.18 (0.26) | 5.18* (.015) | 0.13 (.000) | 0.45 (.003) | 0.69 (.004) |
| **Chocolate** | 3.07 (0.24) | 3.80 (0.25) | 2.86 (0.25) | 2.84 (0.25) | 3.29 (0.27) | 3.38 (0.25) | 0.04 (.000) | 0.11 (.000) | 2.81 (.017) | 2.13 (.013) |
| **Relative unhealthy snack choice** | 0.15 (0.05) | 0.20 (0.05) | 0.19 (0.05) | 0.12 (0.05) | 0.17 (0.06) | 0.18 (0.05) | 2.20 (.007) | 0.43 (.001) | 0.64 (.004) | 0.02 (.000) |
| **Restaurant meal intentions** | | | | | | | | | | |
| **Mac and cheese** | 3.28 (0.27) | 3.21 (0.27) | 3.25 (0.27) | 3.29 (0.27) | 3.84 (0.29) | 3.29 (0.27) | 0.06 (.000) | 1.05 (.003) | 0.53 (.003) | 0.78 (.005) |
| **Salmon and rice** | 4.66 (0.28) | 4.70 (0.28) | 4.77 (0.28) | 4.64 (0.28) | 4.40 (0.31) | 5.15 (0.28) | 0.16 (.000) | 0.01 (.000) | 1.15 (.007) | 0.70 (.004) |
| **KFC® fried chicken** | 2.22  (0.24) | 2.64 (0.24) | 2.79 (0.24) | 2.23 (0.24) | 2.43 (0.26) | 2.44 (0.24) | 3.28 (.010) | 0.84 (.003) | 1.43 (.009) | 0.27 (.002) |
| **Tofu tacos** | 4.27 (0.29) | 3.75 (0.29) | 4.19 (0.29) | 3.81 (0.29) | 4.77 (0.32) | 4.31 (0.29) | 0.11 (.000) | 0.89 (.003) | 0.35 (.002) | 3.18* (.019) |
| **Relative unhealthy restaurant choice** | 1.82 (0.13) | 1.89 (0.13) | 1.90 (0.13) | 1.81 (0.13) | 1.85 (0.14) | 1.71 (0.13) | 0.15 (.000) | 0.55 (.002) | 0.17 (.001) | 0.27 (.002) |
| **Junk food components intentions** | | | | | | | | | | |
| **Sugar** | 2.41 (0.22) | 2.31 (0.22) | 2.40 (0.23) | 2.31 (0.23) | 2.28 (0.25) | 2.28 (0.22) | 5.49* (.016) | 0.21 (.001) | 0.04 (.000) | 0.02 (.000) |
| **Salt** | 2.45 (0.21) | 1.93 (0.21) | 2.37 (0.21) | 1.63 (0.21) | 1.88 (0.23) | 1.86 (0.21) | 16.39** (.047) | 6.96** (.021) | 0.50 (.003) | 1.63 (.010) |
| **Fat** | 2.07 (0.22) | 2.33 (0.22) | 2.43 (0.22) | 1.74 (0.22) | 1.86 (0.24) | 1.72 (0.22) | 8.56** (.025) | 7.87** (.023) | 0.46 (.003) | 0.39 (.002) |

**p* < .05

***p* < .01.

**S5 Table. Comparisons of motivations to eat unhealthy food across conditions while controlling for gender.**

| **Variable** | **Condition** | | | | | | **F_gender_ (η_p_^2^)** | **F_norm_ (η_p_^2^)** | **F_group support_ (η_p_^2^)** | **F_int_ (η_p_^2^)** |
| --- | --- | --- | --- | --- | --- | --- | --- | --- | --- | --- |
|  | **Pro-unhealthy eating norm** | | | **Anti-unhealthy eating norm** | | |  |  |  |  |
|  | **Collective autonomy support**  ***M*(*SE*)** | **Collective control**  ***M*(*SE*)** | **No support**  ***M*(*SE*)** | **Collective autonomy support**  ***M*(*SE*)** | **Collective control**  ***M*(*SE*)** | **No support**  ***M*(*SE*)** |  |  |  |  |
| **Intrinsic motivation** | 4.96 (0.24) | 4.50 (0.25) | 4.90 (0.24) | 4.68 (0.25) | 4.86 (0.26) | 4.85 (0.24) | 0.20 (.001) | 0.00 (.000) | 0.33 (.002) | 0.85 (.005) |
| **Integrated regulation** | 3.47 (0.22) | 2.83 (0.23) | 2.72 (0.22) | 2.69 (0.22) | 3.19 (0.24) | 2.98 (0.22) | 8.96** (.027) | 0.10 (.000) | 0.56 (.003) | 4.08* (.025) |
| **Identified regulation** | 2.02 (0.16) | 1.62 (0.17) | 1.65 (0.16) | 1.79 (0.17) | 1.51 (0.18) | 1.92 (0.16) | 12.55** (.037) | 0.02 (.000) | 2.12 (.013) | 1.29 (.008) |
| **Introjected regulation** | 1.51 (0.15) | 1.38 (0.15) | 1.54 (0.15) | 1.65 (0.15) | 1.56 (0.16) | 1.83 (0.15) | 3.10 (.009) | 2.66 (.008) | 1.02 (.006) | 0.13 (.001) |
| **External regulation** | 1.92 (0.15) | 1.52 (0.16) | 1.60 (0.16) | 1.62 (0.16) | 1.89 (0.17) | 1.45 (0.15) | 0.10 (.000) | 0.04 (.000) | 1.32 (.008) | 2.31 (.014) |
| **Amotivation** | 4.15 (0.29) | 4.63 (0.30) | 3.77 (0.30) | 3.71 (0.30) | 3.34 (0.32) | 3.47 (0.29) | 0.91 (.003) | 7.60** (.023) | 0.87 (.005) | 1.53 (.009) |

**p* < .05

***p* < .01.

**S6 Table. Comparisons of unhealthy eating intentions across conditions while controlling for BMI.**

| **Variable** | **Condition** | | | | | | **F_BMI_(η_p_^2^)** | **F_norm_ (η_p_^2^)** | **F_group support_ (η_p_^2^)** | **F_int_ (η_p_^2^)** |
| --- | --- | --- | --- | --- | --- | --- | --- | --- | --- | --- |
|  | **Pro-unhealthy eating norm** | | | **Anti-unhealthy eating norm** | | |  |  |  |  |
|  | **Collective autonomy support**  ***M*(*SE*)** | **Collective control**  ***M*(*SE*)** | **No support**  ***M*(*SE*)** | **Collective autonomy support**  ***M*(*SE*)** | **Collective control**  ***M*(*SE*)** | **No support**  ***M*(*SE*)** |  |  |  |  |
| **Snack intentions** | | | | | | | | | | |
| **Fruits** | 4.95 (0.24) | 5.21 (0.25) | 4.75 (0.25) | 5.04 (0.25) | 5.21 (0.27) | 5.45 (0.24) | 6.39* (.020) | 1.70 (.005) | 0.38 (.002) | 1.16 (.007) |
| **Chips** | 2.77 (0.23) | 2.72 (0.24) | 2.71 (0.24) | 2.41 (0.24) | 2.66 (0.26) | 2.65 (0.23) | 0.89 (.003) | 0.69 (.002) | 0.10 (.001) | 0.27 (.002) |
| **Vegetables** | 4.32 (0.25) | 4.82 (0.26) | 4.53 (0.26) | 4.54 (0.26) | 4.54 (0.29) | 4.20 (0.26) | 11.89** (.036) | 0.37 (.001) | 0.77 (.005) | 0.70 (.004) |
| **Chocolate** | 3.02 (0.24) | 3.89 (0.26) | 2.78 (0.25) | 2.87 (0.26) | 3.28 (0.28) | 3.38 (0.25) | 2.08 (.006) | 0.08 (.000) | 3.32* (.020) | 2.83 (.017) |
| **Relative unhealthy snack choice** | 0.15 (0.05) | 0.20 (0.05) | 0.18 (0.05) | 0.13 (0.05) | 0.19 (0.06) | 0.18 (0.05) | 2.80 (.009) | 0.11 (.000) | 0.52 (.003) | 0.02 (.000) |
| **Restaurant meal intentions** | | | | | | | | | | |
| **Mac and cheese** | 3.28 (0.27) | 3.33 (0.28) | 3.26 (0.28) | 3.26 (0.28) | 3.81 (0.30) | 3.28 (0.27) | 0.84 (.003) | 0.50 (.002) | 0.75 (.005) | 0.47 (.003) |
| **Salmon and rice** | 4.68 (0.28) | 4.70 (0.29) | 4.75 (0.29) | 4.70 (0.29) | 4.35 (0.31) | 5.14 (0.28) | 0.60 (.002) | 0.01 (.000) | 1.05 (.007) | 0.80 (.005) |
| **KFC® fried chicken** | 2.22 (0.24) | 2.73 (0.25) | 2.78 (0.25) | 2.28 (0.25) | 2.28 (0.27) | 2.40 (0.24) | 0.85 (.003) | 1.60 (.005) | 1.06 (.007) | 0.63 (.004) |
| **Tofu tacos** | 4.28 (0.29) | 3.97 (0.30) | 4.22 (0.30) | 3.85 (0.30) | 4.79 (0.33) | 4.31 (0.29) | 2.21 (.007) | 0.43 (.001) | 0.56 (.004) | 2.13 (.013) |
| **Relative unhealthy restaurant choice** | 1.83 (0.13) | 1.95 (0.13) | 1.87 (0.13) | 1.83 (0.13) | 1.82 (0.14) | 1.66 (0.13) | 0.86 (.003) | 1.16 (.004) | 0.40 (.003) | 0.36 (.002) |
| **Junk food components intentions** | | | | | | | | | | |
| **Sugar** | 2.43 (0.22) | 2.44 (0.23) | 2.40 (0.23) | 2.36 (0.24) | 2.23 (0.26) | 2.19 (0.23) | 0.24 (.001) | 0.72 (.002) | 0.09 (.001) | 0.06 (.000) |
| **Salt** | 2.46 (0.22) | 2.06 (0.22) | 2.44 (0.22) | 1.68 (0.23) | 1.88 (0.24) | 1.79 (0.22) | 0.48 (.001) | 8.52** (.026) | 0.20 (.001) | 0.95 (.006) |
| **Fat** | 2.10 (0.22) | 2.46 (0.22) | 2.51 (0.22) | 1.83 (0.23) | 1.83 (0.25) | 1.65 (0.22) | 1.63 (.005) | 9.88**  (.030) | 0.32 (.002) | 0.89 (.006) |

**p* < .05

***p* < .01.

**S7 Table. Comparisons of motivations to eat unhealthy food across conditions while controlling for BMI.**

| **Variable** | **Condition** | | | | | | **F_BMI_ (η_p_^2^)** | **F_norm_ (η_p_^2^)** | **F_group support_ (η_p_^2^)** | **F_int_ (η_p_^2^)** |
| --- | --- | --- | --- | --- | --- | --- | --- | --- | --- | --- |
|  | **Pro-unhealthy eating norm** | | | **Anti-unhealthy eating norm** | | |  |  |  |  |
|  | **Collective autonomy support**  ***M*(*SE*)** | **Collective control**  ***M*(*SE*)** | **No support**  ***M*(*SE*)** | **Collective autonomy support**  ***M*(*SE*)** | **Collective control**  ***M*(*SE*)** | **No support**  ***M*(*SE*)** |  |  |  |  |
| **Intrinsic motivation** | 4.96 (0.24) | 4.60 (0.26) | 4.91 (0.25) | 4.75 (0.25) | 4.83 (0.27) | 4.84 (0.24) | 0.73 (.002) | 0.01 (.000) | 0.23 (.001) | 0.39 (.003) |
| **Integrated regulation** | 3.38 (0.22) | 2.90 (0.24) | 2.73 (0.23) | 2.74 (0.23) | 3.10 (0.25) | 2.93 (0.22) | 0.52 (.002) | 0.20 (.001) | 0.57 (.004) | 2.32 (.015) |
| **Identified regulation** | 2.00 (0.17) | 1.65 (0.18) | 1.65 (0.17) | 1.79 (0.17) | 1.46 (0.19) | 1.88 (0.17) | 0.04 (.000) | 0.17 (.001) | 1.93 (.012) | 1.03 (.007) |
| **Introjected regulation** | 1.51 (0.15) | 1.41 (0.16) | 1.55 (0.15) | 1.64 (0.15) | 1.46 (0.16) | 1.83 (0.15) | 0.29 (.001) | 1.55 (.005) | 1.35 (.009) | 0.28 (.002) |
| **External regulation** | 1.93 (0.16) | 1.57 (0.17) | 1.60 (0.16) | 1.60 (0.16) | 1.90 (0.18) | 1.46 (0.16) | 0.18 (.001) | 0.14 (.000) | 1.31 (.008) | 2.08 (.013) |
| **Amotivation** | 4.15 (0.29) | 4.67 (0.31) | 3.78 (0.30) | 3.69 (0.31) | 3.27 (0.33) | 3.47 (0.29) | 0.07 (.000) | 8.42** (.026) | 0.79 (.005) | 1.78 (.011) |

**p* < .05

***p* < .01.

**References Not Present in the Manuscript**

1b. Thompson ER. Development and validation of an internationally reliable short-form of the positive and negative affect schedule (PANAS). Journal of Cross-Cultural Psychology. 2007 Mar;38(2):227-42. doi: 10.1177/0022022106297301

2b. Ryan RM, Frederick C. On energy, personality, and health: Subjective vitality as a dynamic reflection of well‐being. Journal of Personality. 1997 Sep;65(3):529-65. doi: 10.1111/j.1467-6494.1997.tb00326.x

3b. Marteau TM, Bekker H. The development of a six‐item short‐form of the state scale of the Spielberger State—Trait Anxiety Inventory (STAI). British Journal of Clinical Psychology. 1992 Sep;31(3):301-6. doi: 10.1111/j.2044-8260.1992.tb00997.x

4b. Spilberger C. Manual for the state‐trait anxiety inventory: STAI (Form Y). Palo Alto (CA): Consulting Psychologists Press; 1983.

5b. Robins RW, Hendin HM, Trzesniewski KH. Measuring global self-esteem: Construct validation of a single-item measure and the Rosenberg Self-Esteem Scale. Personality and social psychology bulletin. 2001 Feb;27(2):151-61. doi: 10.1177/0146167201272002

6b. American Psychiatric Association. Diagnostic and statistical manual of mental disorders. 5th ed. Arlington (VA): American Psychiatric Publishing; 2013.

7b. Emanuel AS, McCully SN, Gallagher KM, Updegraff JA. Theory of planned behavior explains gender difference in fruit and vegetable consumption. Appetite. 2012 Dec 1;59(3):693-97. doi: 10.1016/j.appet.2012.08.007

8b. Dunford EK, Popkin B, Ng SW. Junk food intake among adults in the United States. The Journal of Nutrition. 2022 Feb;152(2):492-500. doi: 10.1093/jn/nxab205

9b. Howarth NC, Huang TT, Roberts SB, Lin BH, McCrory MA. Eating patterns and dietary composition in relation to BMI in younger and older adults. International Journal of Obesity. 2007 Apr;31(4):675-84. doi: 10.1038/sj.ijo.0803456
